# Health Workers Perceptions of Quality in Mental Healthcare at District Hospitals in Johannesburg, South Africa

**DOI:** 10.3390/healthcare14091190

**Published:** 2026-04-29

**Authors:** Makgandeni Libby Pholofolo, Bernard Hope Taderera

**Affiliations:** Department of Environmental Health, Faculty of Health Sciences, University of Johannesburg, Johannesburg 2028, South Africa; libby.mashaba@gauteng.gov.za

**Keywords:** perceptions, quality, mental healthcare, health workers, Johannesburg, district hospitals, South Africa

## Abstract

Background: Mental healthcare has emerged as a major public health issue in the aftermath of COVID-19 worldwide due to global health system challenges which hinder effective healthcare. In this, there is a knowledge gap on research exploring the perceived quality of mental healthcare amongst hospital-based health workers with a particular focus on knowledge and practice, organization and system, and job satisfaction factors for an insight towards strengthening ongoing effort for the realization of the universal health coverage goal of the comprehensive global mental health action plans. The aim of this study was to assess health workers’ perceptions of quality in mental healthcare at three district hospitals in Johannesburg, South Africa. Methods: An exploratory cross-sectional research design was used on a stratified random sample of 160 health workers recruited as participants at the three selected hospitals in Johannesburg. Data were collected using a self-administered questionnaire and then subjected to descriptive statistical analysis using SPSS Version 29. Results: It was established that healthcare workers’ at the three district hospitals in Johannesburg were generally familiar with mental health guidelines and mental disorders which resulted in better patient engagement and prioritisation of mental health as being important as physical health. However the majority of these healthcare workers perceived the quality of mental healthcare at the three hospitals was low. Further assessment however revealed that these perceptions may have emanated from organizational and system incapacity, and limited satisfaction with compensation and benefits, recognition for work done and limited training. Conclusions: Health worker perceptions of quality in mental healthcare help provide an insight into what health systems may need to address mental health service delivery. The study of the three hospitals in Johannesburg, South Africa underscore the need to reinforce knowledge sharing through healthcare worker training, strengthen organisational and system capacity, provide adequate remuneration and benefits, and reinforce clear referral pathways and collaboration with specialists for the realisation of quality improvement and sustenance in pursuing the universal health coverage goal of the WHO Comprehensive Mental Health Action Plans and the Sustainable development Agenda on health of 2030 and beyond.

## 1. Introduction

Mental health in the post COVID-19 pandemic period has emerged as an important public healthcare policy issue for health systems across the world [1]. In Sub-Saharan Africa (SSA), mental health challenges are increasingly being realized as a growing public health concern [2]. South Africa for example faces a severe mental health crisis, with approximately 30% of the population reportedly experiencing a mental health disorder in their lifetime [3]. Despite this high prevalence in this country, mental healthcare remains largely inaccessible, especially in public healthcare settings, which cater for over 85% of the population [4]. In this, South Africa has an estimated treatment gap of 75% for common mental disorders such as anxiety, depressive and substance use disorders. This treatment gap level resonates with the 76–85% for serious mental disorders that have been reported in Low-and Middle-Income Countries (LMICs). A number of factors have been attributed to Africa’s high mental illness treatment gap, and these include low priority at a policy level, lack of skilled personnel, limited financing, and poor allocation of financial resources towards institutional care [5]. In addition, the levels of public expenditure on mental health are low, with a global average of 2.1% of government health expenditure, and particularly lower in LMICs [6]. In South Africa, inadequate finance and inequitable resource distribution were major difficulties even before the COVID-19 pandemic, with mental health receiving only 5% of the health budget despite the high disease burden [4]. Furthermore, even though the average number of mental health workers at a global level is 13 per 100,000 of the population, LIMCs only have about 2 mental healthcare workers per 100,000 [6]. Additionally, cultural beliefs and stigma often prevent individuals from seeking mental health treatment, leading to delayed diagnosis and inadequate care [7]. Mental health experts have consistently stated that the lack of implementation plans, staff shortages, and poor mental health financing pose the most significant challenges undermining the quality of mental healthcare services [8].

Studies have reported factors that may help strengthen the quality of mental healthcare services in primary healthcare settings. A study on Nepal for example, reported mental health service quality factors include the availability of guidelines, protocols and awareness raising materials, provision of supervision, referral systems being in place, patient record keeping, community sensitizations and home visits, and provision of psychosocial counseling. The Nepal study also identified barriers such as the shortage of psychotropic medicines, lack of private space for counseling, workload and health workers’ grievances regarding incentives, and perceived stigma causing dropouts [9]. Another study carried out in Cambodia established that quality in mental health services may require the development of a national mental health policy and strategy to help guide interventions on education, training and resource mobilization and allocation as necessary. This Cambodia study also revealed that addressing mental health stigma and discrimination, raising awareness and understanding of the mental health disease may help empower people with knowledge on the causes and available treatment for mental disorders, to enable people to seek and provide appropriate care and support. The study of Cambodia also noted the importance of strengthening collaboration between mental healthcare stakeholders to help address the lack of trust, personal conflicts, differences in priorities between individuals and organisations and issues around status and professionalism to enable multidisciplinary and holistic services, and integrated system of services for greater coordination of care across the village, district and provincial levels. Other reported factors that may help improve the quality of mental healthcare included the need for mental health services appropriate for the Cambodian culture and context, improving the quality of mental health training and service provision for ethical and professional healthcare. However, this study also reported challenges which include inadequate funding and human resources, and the already overburdened health system as barriers that may undermine quality strengthening for mental health services in Cambodia [10].

However, in pursuing quality in mental healthcare, there is a knowledge gap on research exploring hospital-based healthcare workers’ perceptions of quality in mental healthcare in a public health system. To address this knowledge gap, our study sought to investigate health workers’ perceptions of quality in mental healthcare with a particular focus on knowledge and practice, organizational, and job satisfaction factors at district hospitals in Johannesburg, South Africa.

## 2. Materials and Methods

### 2.1. Research Design and Context

The research design used in this study was cross-sectional and exploratory [11]. This paper was generated from a wider research conducted in three district hospitals in Johannesburg, South Africa, namely Lenasia South, Bheki Mlangeni, and Southrand, in a study titled: Factors associated with the provision of quality mental healthcare among healthcare workers in Johannesburg District Hospitals, South Africa.

### 2.2. Study Population

The participants in this study included healthcare workers directly involved in the provision of mental healthcare at the three Johannesburg District Hospitals. This comprised Medical Doctors (General Practitioners and Specialists such as Psychiatrists), Nurses (General and Psychiatric), Psychologists, Social Workers, Occupational Therapists, Pharmacists, and other Allied Health Professionals who work with mental health patients. The population included employees from a variety of departments, including psychiatric wards, general wards, emergency departments, outpatient clinics, and community outreach programs [11].

### 2.3. Sample Size Determination

**Figure 1:**
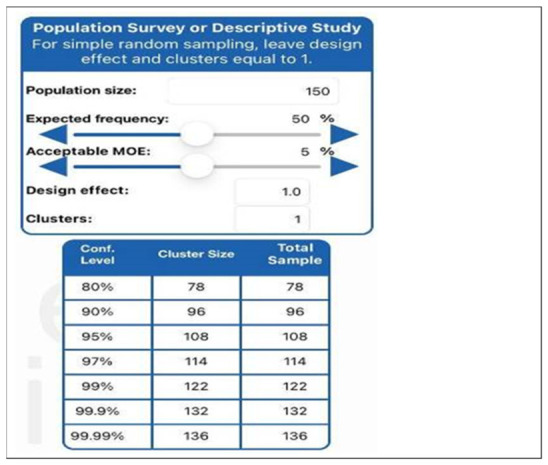
Sample size calculation.

The sample size estimate for the main study was computed using Epi Info Version 7 as shown in Figure 1, with an estimated population size of 150 healthcare workers from the three selected district hospitals.

The error margin of 5% was used at 95% confidence interval, with a 25% contingency of 27. The final calculated sample size was 108 healthcare workers from the three district Johannesburg hospitals. However, we envisaged to collect data from 135 respondents to cater for the possibility of some potential participants failing to complete questionnaires.

### 2.4. Sampling Procedure

A stratified sample strategy was used to assure representation from diverse kinds of healthcare personnel involved in providing mental health care in Johannesburg District Hospitals. This method was advantageous as it minimized selection bias and enhanced the representativeness of the sample across different healthcare professions. A list of all healthcare workers engaged in mental health care in selected hospitals were obtained and stratified by profession (doctors, nurses, psychologists, social workers). Using a random number generator, a predetermined number of participants was selected from each stratum, proportional to their representation in the overall healthcare workforce. This approach ensured that the sample accurately reflects the composition of mental health care providers, facilitating generalization of the findings [11,12].

### 2.5. Data Collection Procedures

A self-administered standardized questionnaire developed from literature review findings was used for primary data collection because it enabled the collection of standardized data from healthcare workers efficiently and cost-effectively [11]. Non-responses were replaced through sampling to select alternative participants. The questionnaire consisted of sections which included demographic information, experiences in providing mental health care, perceived factors affecting care quality, resource availability and training needs. However, given that the data collection tool was not validated, a pilot study on a sample of 10 interviews was carried out to pre-test the data collection tool for validity and reliability. Findings from the pilot study were, however, not included in the final dataset as they helped improve the data collection tool further.

For purposes of this paper however, we were interested in sections focusing on measuring the perceptions of quality as either high or low. In addition, we were also interested in sections in which the Lickert Rating Scale was used to perception levels for knowledge and practice factors which included appropriateness of training, familiarity with guidelines, understanding of mental disorders, confidence in mental healthcare provision, training, patient engagement, opportunity seeking to learn more about mental health, and mental health being valued the same as physical health. In addition, we were interested in questions assessing perception levels for organizational factors which included hospital infrastructure for mental healthcare, adequacy of security, sufficiency of resources, referral pathways and collaboration with mental health specialists, and adequacy of mental health training. Furthermore, we sought data from rated questions assessing perception levels for job satisfaction factors which included satisfaction level, autonomy and decision making, compensation and benefits, feedback and recognition, and hospital recommendation.

To ensure accessibility and inclusion of all potential participants in these districts, the questionnaire was made available only in English as this is the working language in these districts. Research assistants were recruited and underwent comprehensive training to help with questionnaire distribution, collection and ensure standardized administration of the questionnaire by respondents. This training covered the study’s objectives, ethical considerations and proper questionnaire administration techniques. The data-collection process was conducted over a period of two months at the three Johannesburg District Hospitals. After obtaining necessary permissions from hospital management and ethics committees, the research team approached potential participants during their work hours, ensuring minimal disruption to hospital operations. All individuals provided informed consent prior to their involvement. Self-administered questionnaires were distributed in person by trained research assistants who were available to clarify any questions. Participants were given the option to complete the questionnaire immediately or return it within a week to a secure drop-box located in each hospital department. To maximise response rates, two reminder emails were sent to all potential participants at one-week intervals. For night shift workers, special arrangements were made to ensure their inclusion in the study. All completed questionnaires were collected daily from the drop-boxes by the research team and securely stored.

### 2.6. Data Analysis

Descriptive statistical analysis was performed using SPSS Version 29. In this, assessment scores from Likert Rated questions on perceptions of quality focusing on knowledge and practice, organizational, and job satisfaction factors [11].

## 3. Results

### 3.1. Summative Profile of Study Sites and Participants

Data collection was carried out at Lenasia South District Hospital, situated in southern Johannesburg and has approximately 20 beds for mental health care users and primarily serves Indian and Black communities. Fieldwork was also done at Bheki Mlangeni Hospital, located in Soweto, catering for Zulu, Xhosa, Tswana, and Sotho populations, with about 30 beds for mental health patients. Additional research was carried out at Southrand Hospital, in southern Johannesburg, which serves a mixed population including Blacks, Indians, and Whites, offering around 35 beds for mental health care.

In this, a total of 160 participants who were the actual eligible population were selected using the stratified random sampling technique, approached, consented and took part in this study, and completed the questionnaire. These participants were selected due to the higher-than-expected response rate because of increased participant interest, and also due to the fact that we had envisaged that some would not be able to complete the questionnaire which may have necessitated replacements in order for our sample size to be high enough for statistical power. The majority of participants were aged 31–40 years (36.25%: *n* = 58), followed by those aged 41–50 years (31.88%: *n* = 51) as shown in Table 1. Smaller percentages were in the age groups 20–30 years (13.12%: *n* = 21), 51–60 years (15.62%: *n* = 25), and 61+ years (3.12%: *n* = 5). In terms of gender, the majority were female (63.75%: *n* = 102), followed by male participants (34.38%: *n* = 55), with a small proportion preferring not to disclose their gender (1.88%: *n* = 2). Regarding marital status, the majority were single (46.88%: *n* = 75), followed by married participants (38.75%: *n* = 62), and smaller groups in divorced (6.25%: *n* = 10), widowed (5.62%: *n* = 9), and separated (2.50%: *n* = 4) categories. The majority held certificates or diplomas (58.12%: *n* = 93), followed by those with bachelor’s or honours degrees (34.38%: *n* = 55), with smaller proportions holding master’s degrees (5.62%: *n* = 9) or a PhD (1.88%: *n* = 3).

The stratified random sampling yielded a majority Professional Nurses with basic Psychiatry (21.88%, *n* = 35) and General Professional Nurses (15%: *n* = 24), followed by Doctors (11.88%: *n* = 19). Other roles included Enrolled Nurse (13.12%: *n* = 21), Nursing Auxiliary (14.38%: *n* = 23), with the least representation from Psychiatrists (1.25%: *n* = 2). Regarding experience with mental health care users, the majority had worked for 2–5 years (31.25%: *n* = 50), followed by those with 5 years or more of experience (26.88%: *n* = 43), and 1–2 years of experience (20%: *n* = 32). Fewer had worked between 6 months and 1 year (15.62%: *n* = 25), with the least experience being less than 6 months (6.25%: *n* = 10).

### 3.2. Perceptions Towards Quality of Mental Healthcare

**Figure 2:**
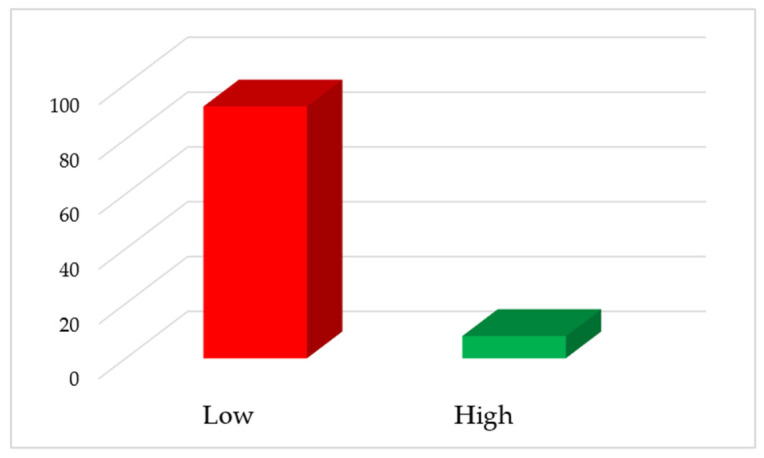
Healthcare worker perceived quality of mental health care provision.

Figure 2 shows the percentage of healthcare workers that perceived quality of mental health care provision from the three district hospitals in Johannesburg, South Africa as either being high or low. The study findings related to the quality of care revealed that the majority of participants perceived the care to be of low quality (91.88%, *n* = 147), while a smaller proportion perceived the care as high quality (8.12%, *n* = 13).

### 3.3. Knowledge, Attitude and Practices

Further assessment of perception levels of quality focusing on knowledge and practice for factors which, as shown in Table 2, included familiarity with guidelines, revealed that the majority of all participants (*n* = 94: 58%) either agreed (*n* = 78: 48%) or strongly agreed (*n* = 16: 10%) that they were familiar with the available mental healthcare guidelines. In comparison, a minority (*n =* 46: 28%) responded either disagree (*n* = 30: 18%) or strongly disagree (*n* = 16: 10%) on this question, with the rest responding neutral, (*n* = 20: 12%) not aware of the availability of guidelines.

As shown in Table 2, the majority health professionals (*n* = 113: 70%) at the three hospitals responded agree (*n* = 88: 55%) and strongly agree (*n* = 25: 15%) in understanding mental health disorders in patients. Only a few (*n* = 20: 11%) revealed that they had attended training on mental healthcare, with (*n* = 14: 8%) responding agree, and (*n* = 6: 3%) strongly agreeing. Despite this however, the majority (*n* = 102: 63%) of health personnel had confidence in the provision of quality mental care in comparison to only few (*n* = 31: 19%) wo responded disagree, and (*n* = 13: 8%) for strongly disagree.

On patient engagement on mental health needs, most (*n* = 56: 35%) of the respondents revealed that they sometimes engaged patients, followed by (*n* = 35: 21%) who responded often, then (*n* = 33: 20%) for always, and (*n* = 14: 8%) never. In addition, the majority (*n* = 58: 36%) revealed that they sometimes sought opportunities to learn more about mental health, followed by (*n* = 29: 18%) who responded always. However, quite a number (*n* = 28: 17%). Most of the health workers revealed that they often (*n* = 69: 43%) and always (*n* = 58: 36%) viewed mental health as being important just like physical health.

### 3.4. Organisational and Systematic Factors

An assessment of the perception levels for organisational and systematic factors was carried out with results outlined in Table 3. On this, the majority of healthcare workers disagreed (*n* = 59: 36%) that hospital infrastructure was conducive to provide mental healthcare. This was followed by an almost similar number (*n* = 57: 35%) who strongly agreed that the hospital infrastructure was conducive for the provision of mental healthcare. In contrast, very few (*n* = 28: 16%) either agreed (*n* = 25: 15%) or strongly agreed (*n* = 3: 1%) that the hospital infrastructure was conducive to provide mental healthcare.

There were somewhat mixed views regarding the adequacy of security where mental healthcare users are catered for with the majority (*n* = 66: 41%) responding agree and quite a number (*n* = 36: 22%) strongly disagreed. In this however, it is important to note that the majority generally agreed (*n* = 84: 52%) with (*n* = 18: 11%) strongly agreeing that there was adequate security where mental healthcare users are cared for. In contrast, the numbers for those who generally disagreed that there was adequate security where mental healthcare users are cared for were lower (*n* = 65: 40%) with a few (*n* = 29: 18%) responding disagree as outlined in Table 3.

Regarding the sufficiency of hospital resources for the provision of mental health services, the majority (*n* = 114: 70%) disagreed, with (*n* = 54: 33%) responding strongly disagree and (*n* = 60: 37%) disagree as shown in Table 3. The findings on hospital provision of adequate mental health training followed a similar pattern with the majority (*n* = 60: 37%) responding disagree, and (*n* = 49: 30% strongly disagree. In comparison, a few (*n* = 24: 14%) agreed that the hospital provided adequate mental health training as evidenced by (*n* = 3: 1%) answering strongly agree and (n = 21: 13%) agree.

The majority health workers who participated in this study (*n* = 46: 28%) agreed that there were clear referral pathways and collaboration with mental health specialists. A closer look however suggested that most participants (*n* = 63: 38%) did not agree with (*n* = 35: 21%) who answered disagree and (*n* = 28: 17%) and (*n* = 28: 17%) responding strongly disagree as outlined in Table 3.

### 3.5. Job Satisfaction Factors

Perceptions were also assessed for job satisfaction as shown in Table 4. In this, it was established that most participants felt satisfied with their work with the majority (*n* = 75: 46%) responding agree and (*n* = 31: 19%) strongly agree. In comparison, only (*n* = 16: 10%) strongly disagreed, and (*n* = 25: 15%) disagreed that they were satisfied with their work. A similar pattern was reported for perceptions regarding autonomy and decision making to provide mental healthcare with the majority (*n* = 68: 42%) responding agree and (*n* = 19: 11%) strongly agree, which was higher when compared to (*n* = 29: 18%) who disagreed and (*n* = 17: 10%) strongly disagreed.

However, it appeared that compensation and benefits were generally not viewed as being competitive not a source of motivation towards the provision of quality mental healthcare. On this, the majority (*n* = 107: 66%) disagreed that compensation and benefits are competitive and motivated them to provide quality mental healthcare with (*n* = 57: 35) responding disagree and (*n* = 50: 31%) strongly disagree. Comparatively, a few (*n* = 31: 19%) agreed, with only (*n* = 8: 5%) answering strongly agree and (*n* = 23: 14%) agree.

Perhaps compounding this was that the majority (*n* = 62: 38%) responded strongly disagree and (*n* = 33: 20%) disagree on the question of whether they received regular feedback and recognition for their work. This was much higher where compared to (*n* = 19: 11%) who responded agreed and only (*n* = 4: 2%) who strongly disagreed. Interestingly, quite a number (*n* = 42: 26%) responded neutral to this question. This pattern also reflected in that the majority (*n* = 79: 49) disagreed that they would recommend their hospital, with (*n* = 48: 30%) who said disagree and (*n* = 31: 19%) strongly disagree. Though the number of healthcare personnel who responded agree (*n* = 49: 30%) was quite sizeable, the cumulative number of those agreeing (*n* = 55: 33%) was comparatively much lower when factoring in the (*n* = 6: 3%) who responded strongly agree.

## 4. Discussion

Knowledge, attitudes and practices are important for quality in mental healthcare. On this, guidelines are a very important source of knowledge for healthcare professionals, which may also enhance positive attitudes and practices in mental health service delivery. Hospital-level Practice Guidelines on Mental Healthcare in South Africa are generally derived from the National Mental Health Policy Framework and Strategic Plan 2023–2030, in the conformity to the Mental Health Act, 2002 (Act No. 17 of 2002), and in line with the World Health Organisation (WHO), which aims to improve mental health through four key strategies focused on achieving: (i.) effectiveness and efficiency in leadership and governance by strengthening capacity through national plans, policies, programmes, and laws for mental health to ensure that they are in line with international human rights standards; (ii) comprehensive, integrated social care services with the goal to move mental healthcare away from large psychiatric hospitals towards community-based settings and the integration of mental health into primary healthcare; (iii) promotion and prevention strategies through the implementation of sector wide interventions with the goal to promote mental well-being and the prevention of mental disorders, focusing specifically on suicide prevention; and (iv) strengthening information systems, evidence and research by enhancing and strengthening the collection and reporting of mental health data which help to inform policies and in progress tracking better [9,13,14,15]. It is commendable and good that healthcare workers at the three district hospitals in Johannesburg, South Africa are generally familiar with these guidelines. This is important considering that clinical practice guidelines are evidence-based and help provide advice on psychotropic prescribing and general mental healthcare, to effectively treat mental health conditions whilst minimising medication and/or treatment adverse effects [16]. However, it is also important to note that guidelines alone may not help improve health workers’ perceptions of quality in mental healthcare.

Regardless however, understanding mental health disorders may help enhance positive perceptions for better treatment and towards enhanced mental health service quality. For example, a study of nursing students at a large metropolitan university in Melbourne, Victoria and Australia reveals that improvement in healthcare workers’ understanding of mental health helps overcome prior knowledge marked by fear and anxiety rooted in cultural beliefs and personal safety concerns. In addition, understanding of mental health conditions help shift the focus to physical symptoms and causality, and eventually to an advanced level of an integrated view recognizing the link between mental and physical health in patients [15]. This underscores the importance of training, which may also help improve confidence in mental healthcare for better quality and treatment outcomes.

Research also emphasizes the importance of patient engagement on mental health needs. Regular patient engagement and communication enhances therapeutic relationships which helps mitigate stigma and boosts client outcomes [6,7]. In addition, healthcare provider engagement significantly improves adherence to treatment and patient satisfaction in mental health contexts [8]. Similarly, the South Africa study of three district hospitals reveals the belief in mental health’s equivalence to physical health as having a positive bearing on the quality care provision. This also reflects a destigmatising mindset and aligns with global mental health advocacy priorities in line with the Comprehensive Mental Health Action Plan set by WHO [9,15,17].

However, the exploratory cross-sectional study of three hospitals in South Africa suggests that negative perceptions of quality in mental healthcare may also originate from incapacities within the organisation and system through which mental healthcare is provided. This study reports that most of the participants (*n* = 116: 71%) view that hospital infrastructure is not conducive for the provision of mental healthcare. In addition, the majority of health workers who participated in this study (*n* = 114: 70%) are of the view that hospital resources are insufficient for the provision of mental health services. This is further compounded by the majority (*n* = 109: 67%) who view mental healthcare training provided by the hospitals as being inadequate. This corresponds with another study which reports that institutional training significantly improves mental health service outcomes in low-resource settings [18,19]. These perceived organisational and systematic factors put into question whether hospitals in the districts of Johannesburg have adequate capacity to contribute towards the realisation of policy outcomes set out in the National Mental Health Policy Framework and Strategic Plan 2023–2030, and the WHO Comprehensive Mental Health Action Plan 2013–2030 for the realisation of the universal health coverage goals of the 2030 Agenda for Sustainable Development [15,17]. Sufficient hospital resources and conducive hospital infrastructure are linked to reduced odds of poor mental health service delivery which highlights the fact that resource adequacy for medication, staff and equipment are vital for the quality of the quality of mental healthcare. This view aligns with a 2018 WHO Report on Barriers to Mental Healthcare, where systemic deficiencies are noted as key hindrances contributing to low quality in mental healthcare [20]. This also mirrors the view in another study which reveals that systemic inadequacies, staff shortages, lack of medication and poor referral systems are key barriers to quality mental health services, especially in Sub-Saharan Africa [21].

Job satisfaction also plays a crucial role in mental health service delivery which has a bearing on the perceptions of health workers towards the quality in mental healthcare. The research on three district hospitals in South Africa reveals that workers who agree that they feel satisfied are significantly more likely to provide quality mental health care. Similarly, those reporting autonomy in mental healthcare decisions achieve better mental healthcare outcomes. This is consistent with Maslach and Leiter’s Burnout Framework of 2016, where autonomy and job satisfaction buffer against burnout, promoting better service delivery [22]. This view confirms findings reported in earlier research which reports a strong link between job satisfaction and patient care quality across healthcare settings [23,24,25].

The study of three district hospitals in South Africa reports that regular feedback and recognition are also significant, especially in the “neutral” and “strongly agree” response categories amongst health workers. Recognition reinforces positive behaviour and increases staff morale, which in turn enhances performance and reinforces positive behaviour [26]. Another study shares this view and reveals that supporting people, supportive environment and supporting learning, and psychological empowerment through feedback mechanisms increases intrinsic motivation and commitment to care quality [27]. Additionally, the research on the three Johannesburg district hospitals in South Africa shows that the willingness to recommend the hospital links strongly with the perceived quality of mental health service provision. This also underscores the fact that workplace reputation influences the extent towhich staff are likely to put extra effort towards recommending their own organisation to the outside world. This reflects the Organizational Citizenship Behaviour (OCB) conceptual framework, where pride in one’s organization motivates high-performance, including compassionate mental health care [28].

### Limitations

Due to financial constraints, we were able to only cover only three hospitals in this study. In addition, the focus of this paper, exploring the perceptions of quality in mental healthcare only provides insights on the subject. Future funded studies may focus on Deepening our Understanding of Quality in Mental Healthcare in South Africa (DUQUMEH-SA) focusing on in district hospitals, and other levels of care, using appropriate statistical models.

## 5. Conclusions

Healthcare workers’ perceptions of quality in mental healthcare help provide an insight into what may need to be reinforced and/or strengthened by health systems in pursuing the universal health coverage goal of the WHO Comprehensive Mental Health Action Plans. It appears that district-level hospitals in Johannesburg, in their endeavour to provide mental healthcare for the realisation of policy outcomes of the National Mental Health Policy Framework and Strategic Plan 2023–2030, are doing well to provide knowledge and clinical practice guidelines and policy documents to their health workforce which is resulting in healthcare worker familiarity with the context of intervening in providing mental healthcare. Familiarity with mental health disorders may contribute towards positive attitudes and perceptions which pave way for responsive mental healthcare of better and high quality.

However, organisational and system incapacities such as inadequate infrastructure and resources are hindrances which undermine mental healthcare and contribute to negative perceptions of quality in mental health service delivery among health professionals. This, compounded by the lack of satisfaction with compensation and benefits, and feedback and recognition may demotivate health workers and undermine the quality of mental healthcare which they provide. However, the fact that health workers in district hospitals in Johannesburg, South Africa are generally satisfied with their work and the level of autonomy and decision to provide mental healthcare that come with it may be a window of opportunity within which to strengthen the organisation and system through which mental health services are provided in order to sustain ongoing efforts towards quality mental healthcare for all in pursuing universal health coverage in a changing world. 

## Figures and Tables

**Table 1:** Study characteristics of healthcare workers.

Characteristic	Category	Frequency (*n* = 160)	Percentage (%)
Age in years	20–30	21	13.12
	31–40	58	36.25
	41–50	51	31.88
	51–60	25	15.62
	61+	5	3.12
Gender	Male	55	34.38
	Female	102	63.75
	Prefer not to say	2	1.88
Marital Status	Single	75	46.88
	Married	62	38.75
	Divorced	10	6.25
	Widowed	9	5.62
	Separated	4	2.50
Level of Education	Certificates/Diploma	93	58.12
	Bachelors/Honours	55	34.38
	Masters	9	5.62
	PhD	3	1.88
Occupational	Doctor general practitioner	19	11.88
	Psychiatrist	2	1.25
	Professional nurse general	24	15.00
	Professional nurse with basic psychiatry	35	21.88
	Professional nurse specialty psychiatry	4	2.50
	Enrolled nurse	21	13.12
	Nursing Auxiliary	23	14.38
	Psychologist	3	1.88
	Social worker	5	3.12
	Occupational Therapist	4	2.50
	Other	20	12.50
Duration of working with mental health care users	less than six 6 months	10	6.25
	6 months–1 year	25	15.62
	1–2 years	32	20.00
	2–5 years	50	31.25
	5 years and above	43	26.88

**Table 2:** Perception levels for knowledge and practice in mental healthcare.

Factors	Category	Frequency (*n* = 160)	Proportion (%)
Familiar with guidelines	Strongly disagree	16	10
	Disagree	30	18
	Neutral	20	12
	Agree	78	48
	Strongly agree	16	10
Understanding mental disorders	Strongly disagree	10	6
	Disagree	24	15
	Neutral	13	8
	Agree	88	55
	Strongly agree	25	15
Confidence in provision of quality mental care	Strongly disagree	13	8
	Disagree	31	19
	Neutral	14	8
	Agree	70	43
	Strongly agree	32	20
Attendance training on Mental healthcare	Strongly disagree	55	34
	Disagree	33	20
	Neutral	52	32
	Agree	14	8
	Strongly agree	6	3
Patient engagement on mental health needs	Never	14	8
	Rarely	22	13
	Sometimes	56	35
	Often	35	21
	Always	33	20
Seeking opportunities to learn more about mental health	Never	23	14
	Rarely	28	17
	Sometimes	58	36
	Often	22	13
	Always	29	18
Mental health is important just like physical health	Never	5	3
	Rarely	9	5
	Sometimes	19	11
	Often	69	43
	Always	58	36

**Table 3:** Perception levels for organizational and systematic factors.

Characteristics	Category	Frequency (*n* = 160)	Proportion (%)
Hospital infrastructure conducive to provide mental health care	Strongly disagree	57	35
	Disagree	59	36
	Neutral	15	9
	Agree	25	15
	Strongly agree	3	1
Adequate security where mental health care users are cared for.	Strongly disagree	36	22
	Disagree	29	18
	Neutral	11	6
	Agree	66	41
	Strongly agree	18	11
Hospital has sufficient resources	Strongly disagree	54	33
	Disagree	60	37
	Neutral	17	10
	Agree	25	15
	Strongly agree	4	2
Clear referral pathways and collaboration with mental health specialists	Strongly disagree	28	17
	Disagree	35	21
	Neutral	44	27
	Agree	46	28
	Strongly agree	7	4
Hospital provision of adequate mental health training	Strongly disagree	49	30
	Disagree	60	37
	Neutral	26	16
	Agree	21	13
	Strongly agree	3	1

**Table 4:** Perception levels for factors relating to job satisfaction.

Characteristics	Category	Frequency (*n* = 160)	Proportion (%)
Feel satisfied with your work	Strongly disagree	16	10
	Disagree	25	15
	Neutral	13	8
	Agree	75	46
	Strongly agree	31	19
Have autonomy and decision making to provide mental health care	Strongly disagree	17	10
	Disagree	29	18
	Neutral	26	16
	Agree	68	42
	Strongly agree	19	11
Compensation and benefits are competitive and motivate me to provide quality mental health care	Strongly disagree	50	31
	Disagree	57	35
	Neutral	22	13
	Agree	23	14
	Strongly agree	8	5
Receive regular feedback and recognition	Strongly disagree	62	38
	Disagree	33	20
	Neutral	42	26
	Agree	19	11
	Strongly agree	4	2
Recommend the hospital (26)	Strongly disagree	31	19
	Disagree	48	30
	Neutral	26	16
	Agree	49	30
	Strongly agree	6	3

## Data Availability

The data presented in this study are available on reasonable request from the corresponding author. The data are not publicly available due to ethical and privacy restrictions involving human participants.

## Abbreviations

The following abbreviations are used in this manuscript:

COVID-19	Coronavirus disease 2019
(DUQUMEH-SA)	Deepening our Understanding of Quality in Mental Healthcare in South Africa
HICs	High Income Countries
KAP	Knowledge, Attitudes and Practices
LMICs	Low and Middle Income Countries
OCB	Organisational Citizenship Behavior
SPSS	Statistical Package for Social Sciences
SSA	Sub-Saharan Africa
WHO	World Health Organisation
